# Indirect excitons in van der Waals heterostructures at room temperature

**DOI:** 10.1038/s41467-018-04293-7

**Published:** 2018-05-14

**Authors:** E. V. Calman, M. M. Fogler, L. V. Butov, S. Hu, A. Mishchenko, A. K. Geim

**Affiliations:** 1 Department of Physics, University of California at San Diego, 9500 Gillman Drive, La Jolla, CA 92093-0319 USA; 2 School of Physics and Astronomy, University of Manchester, Oxford Road, Manchester, M13 9PL UK

## Abstract

Indirect excitons (IXs) are explored both for studying quantum Bose gases in semiconductor materials and for the development of excitonic devices. IXs were extensively studied in III–V and II–VI semiconductor heterostructures where IX range of existence has been limited to low temperatures. Here, we present the observation of IXs at room temperature in van der Waals transition metal dichalcogenide (TMD) heterostructures. This is achieved in TMD heterostructures based on monolayers of MoS_2_ separated by atomically thin hexagonal boron nitride. The IXs we realize in the TMD heterostructure have lifetimes orders of magnitude longer than lifetimes of direct excitons in single-layer TMD and their energy is gate controlled. The realization of IXs at room temperature establishes the TMD heterostructures as a material platform both for a field of high-temperature quantum Bose gases of IXs and for a field of high-temperature excitonic devices.

## Abstract (web summary)

Indirect excitons, composed of a spatially separated electron and hole, could find applications in excitonic devices for signal processing and communication, however they are normally detected at low temperatures. Here, the authors observe room-temperature indirect excitons in van der Waals transition metal dichalcogenide heterostructures.

## Introduction

An indirect exciton (IX) is composed of an electron and a hole confined in spatially separated quantum well layers. Long lifetimes of IXs allow them to cool below the temperature of quantum degeneracy giving the opportunity to create and study quantum Bose gases in semiconductor materials^1,2^. Furthermore, IXs are explored for the development of excitonic devices with energy-efficient computation and seamless coupling to optical communication^3^. IXs were extensively studied in III–V and II–VI semiconductor heterostructures. However, their range of existence has been limited so far to low temperatures due to low IX binding energies in these materials. IXs in van der Waals transition-metal dichalcogenide (TMD) heterostructures^4^ are characterized by high binding energies making them stable at room temperature and giving the opportunity for exploring high-temperature quantum Bose gases in materials and for creating excitonic devices operational at room temperature, the key for the development of excitonic technology^5–7^.

Experimental studies of quantum degenerate Bose gases of IXs were performed so far in GaAs coupled quantum well (CQW) structures where quantum degeneracy was achieved in the temperature range of few Kelvin. The findings include spontaneous coherence and condensation of excitons^8^, long-range spin currents and spin textures^9^, spatially modulated exciton state^10^, and perfect Coulomb drag^11^. Furthermore, IX energy, lifetime, and flux can be controlled by voltage that is explored for the development of excitonic devices. Excitonic devices with IXs were demonstrated so far at temperatures below ~100 K. These devices include traps, lattices, conveyers, and ramps, which are used for studying basic properties of cold IXs, as well as excitonic transistors, routers, and photon storage devices, which hold the potential for creating excitonic signal processing devices and excitonic circuits, a review of excitonic devices can be found in ref. ^3^.

A finite exciton binding energy *E*_ex_ limits the operation temperature of excitonic devices. Excitons exist in the temperature range roughly below *E*_ex_/*k*_B_ (*k*_B_ is the Boltzmann constant)^12^. Furthermore, the temperature of quantum degeneracy, which can be achieved with increasing density before excitons dissociation to electron–hole plasma, also scales proportionally to *E*_ex_^5^. These considerations instigate the search for material systems where IXs have a high-binding energy and, as a result, can provide the medium for the realization of high-temperature coherent phenomena and excitonic devices.

IXs were explored in various III–V and II–VI semiconductor QW heterostructures based on GaAs^2,3,8–14^, AlAs^15,16^, InGaAs^17^, GaN^18–20^, and ZnO^21,22^. Among these materials, IXs are more robust in the ZnO structures where their binding energy is about 30 meV^21^. Proof of principle for the operation of IX switching devices was demonstrated at temperatures up to ~100 K in AlAs/GaAs CQW^16^ where the IX binding energy is about ~10 meV^15^. Studies of IXs in III–V and II–VI semiconductor materials continue to attract intense interest.

Van der Waals structures composed of atomically thin layers of TMD offer an opportunity to realize artificial materials with designable properties, forming a new platform for studying basic phenomena and developing optoelectronic devices^4^. TMD heterostructures allow IXs with remarkably high binding energies^5–7^, much higher than in III–V or II–VI semiconductor heterostructures. Therefore, IXs in TMD heterostructures open the opportunity to realize room-temperature excitonic devices and explore high-temperature quantum degenerate Bose gases of IXs.

The experimental approaches to the realization of IXs in TMD materials involve two kinds of heterostructures. In type I TMD heterostructures with direct gap alignment, the electron and hole layers are spatially separated by a barrier layer. Such type I structures with MoS_2_ forming the QW layers and a hexagonal boron nitride (hBN) forming the barrier were considered in refs. ^5,23^. These heterostructures are similar to GaAs/AlGaAs CQW heterostructures where GaAs forms QW layers and AlGaAs forms the barrier^2,3,8–14^. In type II TMD heterostructures with staggered band alignment, the electron and hole layers form in different adjacent TMD materials such as single-layer MoSe_2_ and WSe_2_^24–27^, MoS_2_ and WSe_2_^28,29^, MoS_2_ and WS_2_^30,31^, MoSe_2_ and WS_2_^32^, and MoS_2_ and MoSe_2_^33^. These heterostructures are similar to AlAs/GaAs CQW heterostructures where electrons and holes are confined in adjacent AlAs and GaAs layers, respectively^15,16^.

Here, we report on the realization of IXs in TMD heterostructures at room temperature. This was achieved using the previously demonstrated approach with MoS_2_/hBN type-I CQW^23^ combined with an improved structure design and detected using time-resolved optical spectroscopy.

**Fig. 1:**
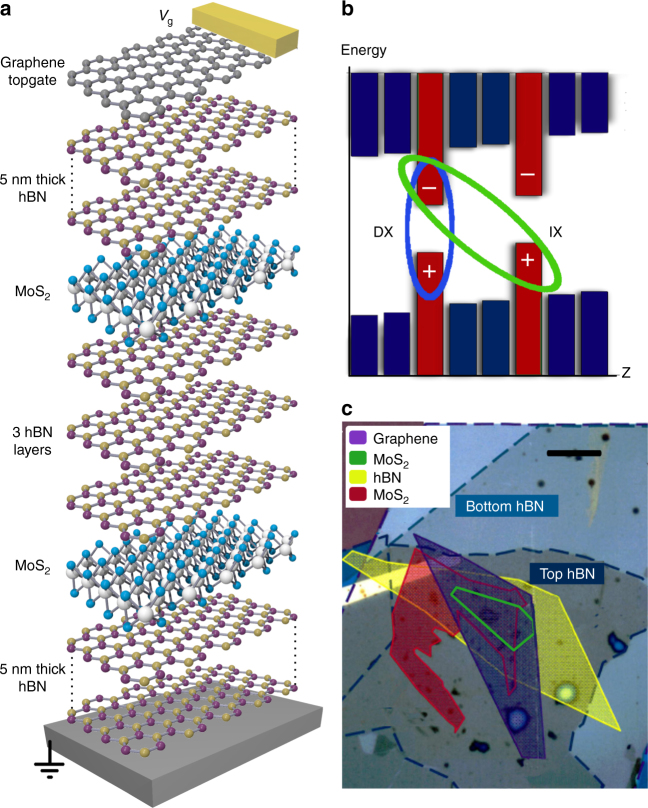
MoS_2_/hBN coupled quantum well heterostructure. The coupled quantum well van der Waals heterostructure layer (**a**) and energy-band (**b**) diagrams. The ovals indicate a direct exciton (DX) and an indirect exciton (IX) composed of an electron (−) and a hole (+). (**c**) Microscope image showing the layer pattern of the device, scale bar is 10 μm

The structure studied here was assembled by stacking mechanically exfoliated two-dimensional crystals on a graphite substrate, which acts as a global backgate (Fig. 1a). The top view of the device showing the contours of different layers is presented in Fig. 1c. The CQW is formed where the two MoS_2_ monolayers, separated by three hBN layers, overlap. IXs are formed from electrons and holes in different MoS_2_ layers (Fig. 1b). The top and bottom 5 nm thick hBN serve as dielectric cladding layers. Voltage *V*_g_ applied between the top graphene layer and the backgate is used to create the bias across the CQW structure. The thickness of hBN cladding layers is much smaller than in our previous CQW TMD device^23^. This allowed us to achieve a much higher electric field across the structure for the applied voltage and, in turn, realize effective control of IX energy by voltage as described below.

## Results

### Long-lifetime emission

**Fig. 2:**
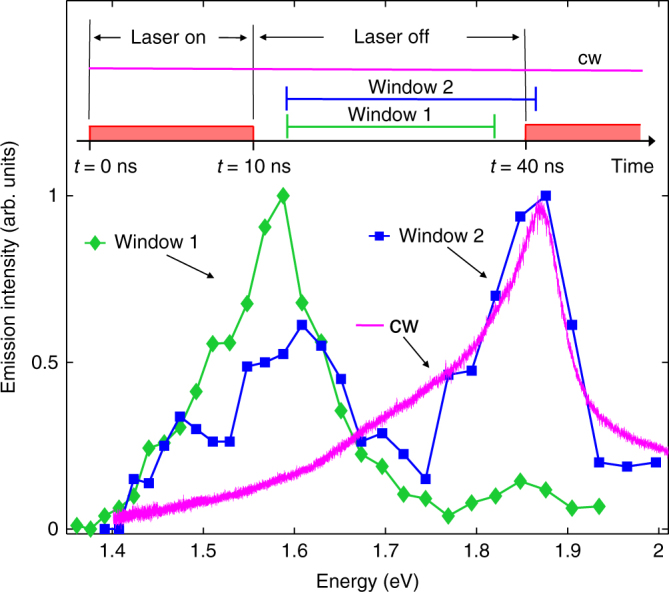
Emission spectra. Spectrum taken in a time-integration window after the laser pulse (window 1) when most of short-lifetime DXs recombine (green diamonds). The spectrum shows emission of long-lifetime IXs. The laser profile and signal integration windows are shown above. The laser has a pulse duration of 10 ns and a period of 40 ns. Window 2 presents emission from a combination of the laser off and the laser on in a ratio that shows both IX and DX (blue squares). cw spectrum (magenta line) is dominated by direct recombination. *T* = 300 K. *V*_g_ = 0

IXs dominate the emission spectrum measured after the laser excitation pulse (Fig. 2). At the time delays exceeding the DX recombination times, most of DXs have recombined, and so the recombination of IXs, which have a much longer lifetime, is not masked by the DX recombination. Both short-lifetime DX and long-lifetime IX emission lines are observed in the spectrum measured in the time window between the laser pulses and the first ≈2 ns of the laser pulse (Fig. 2). As the fraction of time corresponding to the laser pulse grows, the relative intensity of the DX emission increases. In the cw regime, where the laser is permanently on, DXs dominate the spectrum due to their higher oscillator strength (Fig. 2). Supplementary Figure 2 shows similar spectra at 2 K (Supplementary materials present kinetics at different *V*_g_ and low-temperature spectra).

**Fig. 3:**
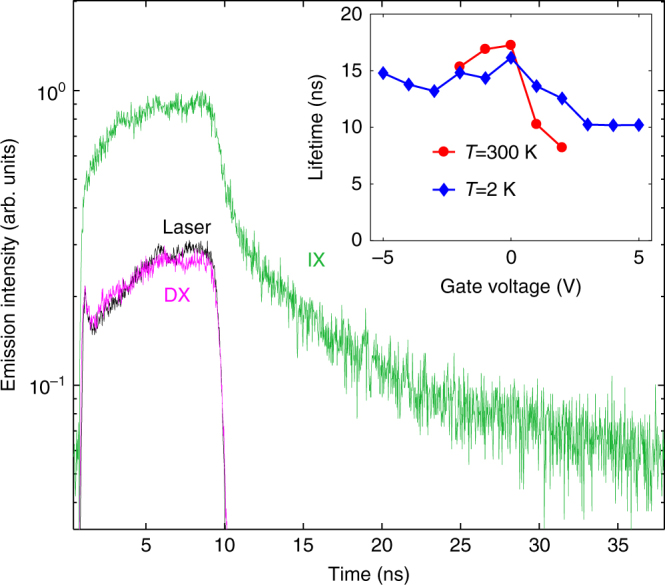
Emission kinetics. Emission kinetics at energies of 1.46–1.65 eV corresponding to the IX spectral range (green), 1.89 eV corresponding to the DX spectral range (magenta), and 3.07 eV corresponding to the excitation laser (black) at *T* = 300 K and *V*_g_=0. IX lifetimes are shown in the inset as a function of gate voltage *V*_g_ for *T*=2 and 300 K, see text. The laser excitation has a pulse duration of 10 ns and a period of 40 ns

The IX emission kinetics is presented in Fig. 3. The time resolution of the experimental system including the pulse generator, the laser, the photomultiplier, and the time-correlated photon counting system is ~0.5 ns as seen from the laser decay kinetics measured at *E*_ex_=3.07 eV. The DX decay kinetics measured at the DX line peak *E*_DX_=1.89 eV closely follows the excitation laser decay indicating that the DX lifetime is shorter than the 0.5 ns experimental resolution. The decay kinetics in the IX spectral range 1.46–1.65 eV shows a double-exponential decay (Fig. 3). Its faster component is determined by the decay of low-energy DX states, which appear in the IX spectral range due to the spectral broadening of the DX line (Fig. 2) (similar localized DXs at low energies in the spectral range of IXs were studied in GaAs/AlAs CQW in ref. ^34^). The slower component is determined by the IX decay (Fig. 3). The IX lifetime ~10 ns (Fig. 3) is orders of magnitude longer than the DX lifetime^35^ and is controlled by gate voltage *V*_g_ over a range of several ns (Fig. 3, inset). The voltage dependence of the IX lifetime has two characteristic features. First, it reduces at positive *V*_g_ where the IX energy approaches the DX energy (section: “Control of Energy by Voltage”). Second, it has a local maximum around *V*_g_=0 (Supplementary Fig. 1) (Supplementary materials present kinetics at different *V*_g_ and low-temperature spectra). Both these features are characteristic of IXs^16^. The former can be attributed to the increase of the overlap of electron and hole wave functions with approaching the direct regime. The latter may result from the suppression of the leakage currents through the CQW layers at zero bias. The realization of the indirect regime, where the IXs are lower in energy than DXs, already at *V*_g_=0 indicates an asymmetry of the device, presumably due to unintenional doping.

### Transport

**Fig. 4:**
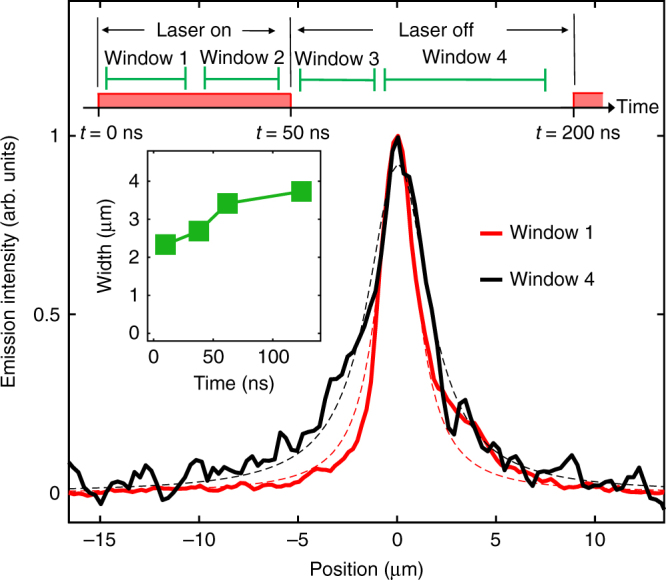
Spatial profiles of emission. Spatial width of the emission at the IX energy 1.46–1.65 eV measured in time windows shown above. The time evolution of the exciton emission width extracted by fitting to lorentzian profiles (dashed lines) is shown in the inset. The excitation laser has a pulse duration of 50 ns and a period of 200 ns. *T* = 300 K.·*V*_g_ = 0

Figure 4 shows the spatial profiles of IX emission. The width of the emission profiles determined by a fit to Lorentzian distribution (dashed lines in Fig. 4) is shown in inset to Fig. 4 as a function of time. The emission profiles after the laser excitation pulse have wider spatial distributions than during the pulse. During the laser excitation pulse, the low-energy tail of short-lifetime DX emission strongly contributes to the emission in the IX spectral region (Fig. 2). After the pulse, DXs decay quickly and the emission is dominated by the long-lifetime IXs. Diffusion of IXs away from the laser excitation spot during their long lifetime contributes to the wider spatial profiles of IX emission. The increase in emission width after the pulse end *l* can be used for estimating an upper bound on the IX diffusion coefficient *D*. For *l*~1 μm (Fig. 4) and IX lifetime *τ* ~ 10 ns (Fig. 3) this gives *D* *~* *l*^*2*^ / *τ* ~ 1 cm^2^/s.

### Control of energy by voltage

**Fig. 5:**
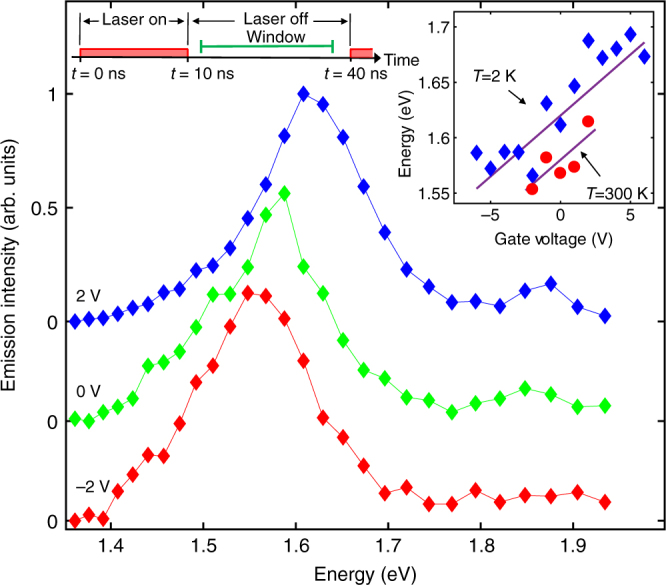
Control of energy by voltage. Spectra at a delay after the laser excitation pulse in the time window shown above at gate voltages *V*_g_=−2, 0, and 2 V at *T*=300 K. Gate voltage dependence of IX energies at *T*=300 and 2 K is shown in the inset. The excitation laser has a pulse duration of 10 ns and a period of 40 ns

Figure 5 shows control of the IX energy by gate voltage. The energy of the long-lifetime emission line shifts by about 120 meV at cryogenic temperatures and by about 60 meV at room temperature. No leakage current or sample damage was detected at cryogenic temperatures in the measurements at applied voltages up to ±6 V, which is typical for thin hBN that can withstand electric fields of about 0.5 V/nm^36^. However, at room temperature, applying ±3 V led to the appearance of leakage current through the device and the reduced device resistivity persisted after lowering *V*_g_. This limited the maximum applied voltage and, in turn, the IX energy shift at room temperature.

## Discussion

Regarding the physical mechanism that governs the IX lifetime in the studied heterostructure we can say the following. In general, this lifetime is limited by tunneling through the hBN spacer. However, direct tunneling across the entire thickness 3 × 0.33=1 nm of the spacer should be prohibitively slow. The tunneling action and tunneling probability can be estimated to be *S* ~ 12 and exp(−2*S*) ~ 10^−11^, respectively, for the potential barrier of height 2 eV and the carrier mass *m*_b_ ~ 0.5 inside the barrier (similar to ref. ^5^). Therefore, we surmise that the IX recombination involves transmission through some midgap defects in the spacer^36^ (specific properties of these defects beyond their ability to facilitate tunneling through the hBN layer, relevant to the experiment, are unclear).

In summary, IXs were observed at room temperature in van der Waals MoS2/hBN heterostructure. The IXs have long lifetimes, orders of magnitude longer than lifetimes of direct excitons in single-layer MoS2, and their energy is controlled by voltage at room temperature.

## Methods

### Experimental setup

The excitons were generated by a semiconductor laser with excitation energy *E*_ex_=3.07 eV. In continuous wave (cw) experiments, photoluminescence (PL) spectra were measured using a spectrometer with resolution 0.2 meV and a liquid-nitrogen-cooled CCD. In all time-resolved experiments, the laser pulses had a rectangular shape with the duration 10–50 ns, period 40–200 ns, and edge sharpness ~0.5 ns (Fig. 2). The laser was focused to a ~2 μm spot with a power of 0.8 mW. In time-resolved PL kinetics and spectrum measurements, the emitted light was filtered by an interference filter or diffracted by the spectrometer, respectively, and then detected by a photomultiplier tube and time-correlated photon counting system. In time-resolved imaging experiments, the emitted light was filtered by an interference filter and detected by a liquid-nitrogen-cooled CCD coupled to a PicoStar HR TauTec time-gated intensifier. The measurements were performed in a ^4^He atmosphere at room temperature (*T*≈300 K) and in a liquid ^4^He cryostat at 2 K.

## Data availability

All relevant data are available from the authors.

## Electronic supplementary material

Supplementary Information
