# Supplementary material for: Indirect excitons in van der Waals heterostructures at room temperature
Source: Nat Commun. 2018 May 14;9:1895. doi: 10.1038/s41467-018-04293-7 (PMC5951911; doi:10.1038/s41467-018-04293-7)
Supplement: Supplementary file 1 — Supplementary Information [file 41467_2018_4293_MOESM1_ESM.pdf]

Supplementary material: Indirect excitons in van der Waals heterostructures at room temperature

E.V. Calman[1], M.M. Fogler[1], L.V. Butov[1] and S. Hu[2], A. Mishchenko[2], A.K. Geim[2]

(Dated: April 8, 2018)

Supplementary materials present kinetics at different V_g and low-temperature spectra.

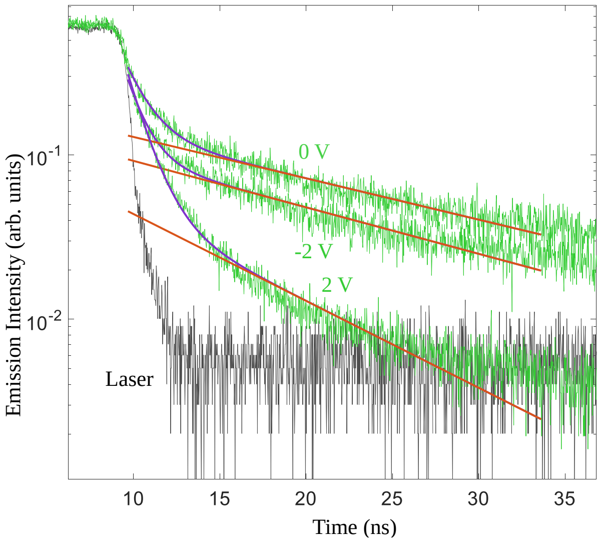

Supplementary Figure 1: Emission kinetics at energies of 1.46–1.65 eV corresponding to the IX spectral range (green) at gate voltages $V_g = -2, 0$, and 2 V at $T = 300$ K. Double exponential fits (blue) and their slower components (red) corresponding to long lifetimes presented in Fig. 3 insert are shown. The laser excitation (black) has a pulse duration of 10 ns and a period of 40 ns.

---

[1] Department of Physics, University of California at San Diego, La Jolla, CA 92093-0319, USA
[2] School of Physics and Astronomy, University of Manchester, Manchester M13 9PL, UK

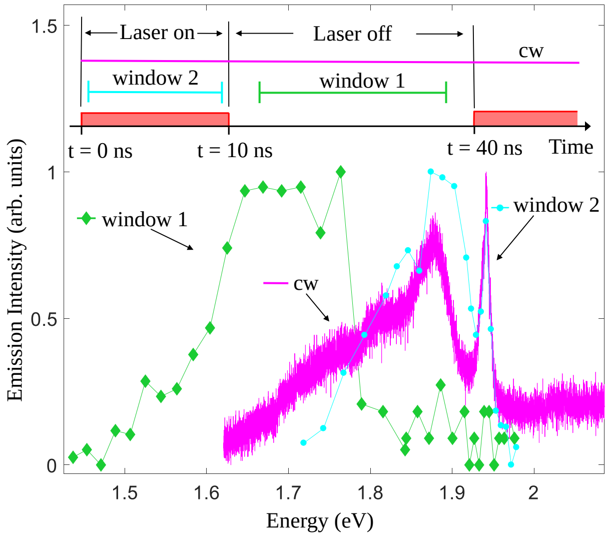

Supplementary Figure 2: Emission spectrum taken in a time-integration window after the laser pulse (window 1) when most of short-lifetime DXs recombine (green diamonds). The spectrum shows emission of long-lifetime IXs. The laser profile and signal integration windows are shown above. The laser has a pulse duration of 10 ns and a period of 40 ns. Emission measured in window 2 during the laser pulse (cyan points) and cw spectrum (magenta line) are dominated by direct recombination. $V_g = -6$ V. $T = 2$ K.
